# The biological function of tumor-derived extracellular vesicles on metabolism

**DOI:** 10.1186/s12964-023-01111-6

**Published:** 2023-06-22

**Authors:** Xiaoyu Fu, Junlong Song, Wei Yan, Bradley M. Downs, Weixing Wang, Juanjuan Li

**Affiliations:** 1 Department of Breast and Thyroid Surgery, Renmin Hospital of Wuhan University, Wuhan, 430060 Hubei China; 2 Department of General Surgery, Renmin Hospital of Wuhan University, Wuhan, 430060 Hubei China; 3 School of Life Science, Wuhan University, Wuhan, 430072 Hubei China; 4 Department of Oncology, Johns Hopkins University School of Medicine, Baltimore, MD 21231 USA; 5 Cancer Center, Renmin Hospital of Wuhan University, Wuhan, 430060 Hubei China

**Keywords:** Extracellular vesicles (EVs), Tumor-derived exosomes (TDEs), Metabolism, Glycolysis, Lipid metabolism

## Abstract

Multiple studies have shown that extracellular vesicles (EVs) play a key role in the process of information transfer and material transport between cells. EVs are classified into different types according to their sizes, which includes the class of exosomes. In comparison to normal EVs, tumor-derived EVs (TDEs) have both altered components and quantities of contents. TDEs have been shown to help facilitate an environment conducive to the occurrence and development of tumor by regulation of glucose, lipids and amino acids. Furthermore, TDEs can also affect the host metabolism and immune system. EVs have been shown to have multiple clinically useful properties, including the use of TDEs as biomarkers for the early diagnosis of diseases and using the transport properties of exosomes for drug delivery. Targeting the key bioactive cargoes of exosomes could be applied to provide new strategies for the treatment of tumors. In this review, we summarize the finding of studies focused on measuring the effects of TDE on tumor-related microenvironment and systemic metabolism.

Video Abstract

**Supplementary Information:**

The online version contains supplementary material available at 10.1186/s12964-023-01111-6.

## Introduction

In the last few years, there has been increased interest in the roles and biological functions of extracellular vesicles (EVs). Exosomes are one type of EVs and are 30-100 nm in diameter [1]. The outer membrane of exosomes is a lipid bilayer that protects the exosome contents from various stimuli in the circulating fluid. This protection allows the exosomes and its contents to achieve long-distance transport in circulating body fluids [2]. Exosomes can contain multiple soluble bioactive substances, such as DNA, RNA, metabolites, lipids and proteins. The specific contents of the exosomes are determined by the cellular source. The content of EVs can be affected by biological factors such as age, gender, and race [3]. Exosome-mediated transfer of DNA, proteins, mRNA and noncoding RNA can lead to the phenotypic change in target cells, which can induce physiological or pathological states [4].

The formation process of exosomes is thought to begin with the establishment of intraluminal vesicles (ILVs), which accumulate in the lumen and then lead to the formation of multivesicular bodies (MVBs). While a proportion of MVBs are degraded by lysosomal fusion, MVBs could also bind to the plasma membrane and could be secreted extracellularly [5]. Exosomes can be secreted into the extracellular environment by a variety of cells, including tumor cells [6], to act on local or distal target cells [7]. Endosomal sorting complex required by ESCRT (endosomal sorting complex required for transport) machinery controls the biogenesis and formation of exosomes. The ESCRT is composed of five different proteins (ESCRT-0, ESCRT-I, ESCRT-II, ESCRT-III and the AAA ATPase Vps4 complex) [8, 9]. In addition to the ESCRT mechanism, sphingolipids ceramide [10], small GTPase ADP ribosylation factor 6 (ARF6) and its effector phospholipase D2 (PLD2) [11], the tetraspanin CD63 in melanocytes [12] can also directly regulate the biogenesis and material sorting of exosomes.

Exosomes act on receptor cells in three main ways: first, proteins on the exosome membrane directly contact with proteins on the receptor cell membrane to trigger intracellular signaling cascades; second, the contents of the exosome membrane are delivered to the receptor cells after fusion with the receptor cell membrane; and third, the target cells directly engulf exosomes [13]. Uptake of exosomes is not random but depends on the interaction between recipient cells and proteins on the surface of exosomes. Some reports have shown that adhesion related molecules on exosomal surfaces determine which cells can receive exosomes. These adhesion molecules include tetraspanins, glycoproteins, and integrins [14, 15]. As a bridge for material and information transfer between cells, exosomes play a key role in local and systemic cancer cell communications. Increasing evidences have shown that exosomes play an important role in tumor proliferation, metastasis, apoptosis, and resistance to drug therapy [16, 17].

In comparison to the normal cell, the tumor cell have an increased consumption of glucose and also undergo metabolism alterations required to sustain growth and reproduction in a limited nutritional environment. Even under oxygen-rich conditions, cancer cells have a much higher rate of glycolysis than tricarboxylic acid (TCA) cycle-mediated oxidative phosphorylation (OXPHOS), a phenomenon known as the “Warburg effect” [18]. Increased glucose uptake and enhanced glycolysis, as well as high lactate production under aerobic conditions, are all considered markers of tumors. The increased demands for lipids and cholesterol in highly proliferating cancer cells also lead to changes in lipid metabolism [19]. Similarly, amino acid metabolism is increased in cancer cells, particularly in the metabolism of the major energy substrates glutamine and serine [20]. Metabolism does not exist independently but acts in concert to provide fertile soil for tumor reproduction and growth.

Studies have shown that in order to survive in the complex tumor microenvironment (TME), tumor cells increase the number of secreted exosomes to actively construct conditions suitable for their growth [21]. Tumor cells release exosomes through various regulatory mechanisms to transmit signals to other cells that trigger subsequent cancer-promoting effects, which include signals that induce invasion, metastasis, angiogenesis, or defensive effects [22]. Exosomes play a key role as a link of information and material transfer between cells in the process of tumor metabolic changes. Therefore, this review focuses on the relationship between tumor-derived exosomes and tumor metabolism, body metabolism, and their impacts on the tumorigenesis and development. It is hoped that this can provide new diagnosis and treatment ideas and strategies for tumors.

## Tumor-derived exosomes

Multiple cell types, including tumor cells, can secret exosomes. The exosomes secreted by tumor cells are called tumor-derived exosomes (TDEs). Studies have estimated that the blood from cancer patients contains twice as many exosomes as the blood from healthy individuals [23]. TDE can transport information and materials not only between tumor cells, but also between stromal cells and tumor cells. Stromal cells receive exosomes from tumor cells and generate a tumor-promoting microenvironment. In turn, exosomes secreted by stromal cells act on tumor cells to promote their proliferation and invasion [24].

Selective protein sorting, controlled by posttranslational modifications, results in the enrichment of certain molecules in specific exosomes [25]. Exosomes from different cells have different characteristics. For example, adiponectin is unique to adipocyte-derived exosomes, while platelet-derived exosomes are rich in leukotrienes and prostaglandins [26]. The contents and receptors expressed by different tumor cells are also variable. For example, exosomes produced in LIM1215 (a colorectal cancer cell line) have higher level of cholesterol, sphingolipids, glycerol, and glycerolipids in comparison to other cell lines [27]. Furthermore, exosomes secreted by prostate cancer cells are rich in phosphatidylserine, high-glucose sphingomyelin, sphingomyelin, and cholesterol [28]. The presence of programmed cell death ligand 1(PD-L1) was found on exosomes isolated from the plasma of patients with non-small cell lung cancer (NSCLC), and its abundance was associated with PD-L1 positivity in tumor tissues [29]. mRNA, miRNA, DNA, and proinflammatory proteins could be enriched in TDEs from lung cancer cells, and with the transfer of these molecules, the proinflammatory phenotype of mesenchymal stem cells (MSCs) is induced and tumor cell proliferation is stimulated [30, 31].

The biogenesis and exocytosis pathways of TDEs are also regulated by specific intracellular mechanisms. The composition, function and possible targeting of exosomes are determined by modulation. Cellular and environmental signals such as inflammation, stress, or cell-cycle events could alter the specific cargo loaded into exosomes or regulate the whole process of exosome release [32]. For example, low pH and hypoxia are important features of TME, both of which promote exosome release and uptake [33, 34]. Acidic environments are best suited for the presence and sequestration of exosomes [35]. Furthermore, it has been shown that low pH can alter the lipid composition in exosome membranes, thereby enhancing exosome uptake by homologous tumor cells [34]. Pyruvate kinase M2 (PKM2) plays a key role in glucose metabolism in cancer cells, and its expression is increased under hypoxia [36]. PKM2 could promote exosome secretion by phosphorylating synaptosome associated protein 23 (SNAP-23) [37]. In summary, the secretion and transport of TDEs in TME may benefit from acidic and hypoxic microenvironment. Recently, Gurunathan et al*.* found that platinum nanoparticles (PtNPs) infused with lutein stimulate the release of exosomes in human lung epithelial adenoma cells (A549) by inducing oxidative stress and the ceramides pathway. The function of PtNPs to promote exosome release provides a new therapeutic target for tumor [38].

In addition to inducing tumorigenesis, TDEs may trigger a parenthetical signaling response at the site of metastasis. It has been proposed that TDEs may induce pre-metastatic niche formation, and thus enhance the progression of cancer metastasis [39]. Some studies have provided evidence that TDEs can inhibit the immune response to target tumors cells, resulting in tumor escape [40]. Furthermore, TDEs have also been found to be an important factor for the promotion of drug resistance. Studies have shown that exosomes of colorectal cancer cells can activate hepatic stellate cells to secrete IL-6. The secretion of IL-6 has been shown to regulate the lactate metabolism of hypoxic tumor cells to confer the resistance of SN38, which is the active metabolite of irinotecan [41]. Furthermore, cisplatin-resistant NSCLC cells transmit drug resistance by promoting glycolysis in sensitive cells through exosome PKM2 [42]. In conclusion, TDEs play an important role in tumor progression, immune escape and drug resistance.

## The effects of exosomes on tumor metabolism

**Fig. 1:**
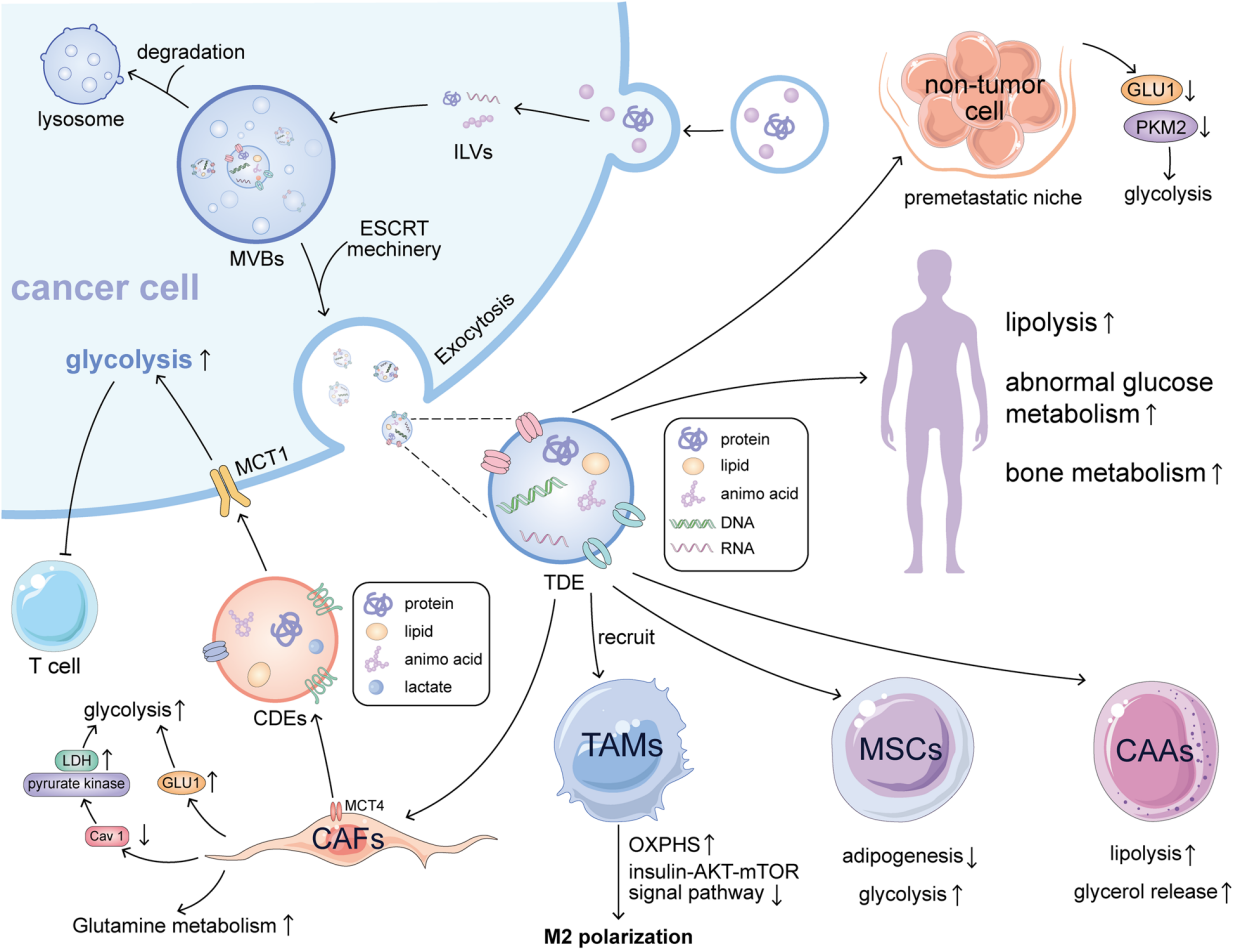
Regulation of metabolism by tumor-derived exosomes. Tumor-derived exosomes (TDEs) carrying specific substances are secreted after intracellular ESCRT machinery. TDEs act on CAFs to promote glycolysis by up-regulating GLU1 expression and down-regulating Cav1 expression. Then CAF-derived exosomes (CDEs) also act in reverse to promote glycolysis of tumor cells. TDEs could promote the polarization of TAMs towards M2 by increasing the level of oxidative phosphorylation (OXPHS) and inhibiting insulin-AKT-mTOR signal pathway of TAMs. TDEs act on MSCs to inhibit adipogenesis and promote glycolysis. TDEs act on CAAs to promote lipolysis and glycerol release. TDEs act on the pre-metastatic niche to reduce glycolysis by reducing the expression of GLU1 and PKM2 in non-tumor cells, creating a more suitable survival environment for metastasis. For the whole body, TDEs improve lipolysis, abnormal glycose metabolism and bone metabolism

Tumor development is dependent on the surrounding environment for nutrients, which often requires the cooperation between cells in TME. TME is a highly heterogeneous and complex ecosystem incorporating both tumor and non-tumor cells. These non-tumor cells include immune and inflammatory cells, bone marrow derived cells, cancer associated fibroblasts (CAFs), adipocytes, endothelial cells and pericytes composing tumor vasculature, and the extracellular matrix (ECM) establishing a complex cross-talk with the tumor [43]. Various components affect tumor metabolism through complex interactions. Due to the remodeling nature of tumor metabolism, it is important to explore the relationship between cellular communication mediators and metabolism. In the TME, communication between cells has been shown to increase in frequency and complexity. TDEs have been shown to be important signal transmitters and are involved in the complex metabolic regulation processes of glucose and lipid metabolism (Fig. 1).

### Glucose metabolism

Glucose metabolism could be divided into anabolism and catabolism. Anabolism refers to the synthesis of glycogen macromolecules from monosaccharides, which requires energy consumption. After a series of decomposition reactions, glucose can release large amounts of energy to the organism. Simultaneously, intermediates formed in the decomposition process can be used as raw materials for the synthesis of biological macromolecules such as proteins, lipids and nucleic acids [18]. Glucose metabolism plays a vital role in communicating the body metabolisms and maintaining the normal life activities. Under abundant oxygen supply, normal cells direct glucose derived pyruvate to mitochondrial oxidative phosphorylation to produce ATP. However, tumor cells generally show greater glucose uptake, lysozyme flux, and lactate secretion regardless of oxygen availability [18]. Moreover, the fundamental roles of TDEs play in this biological process are intriguing.

CAFs are important components of TME. The dynamic interaction between CAFs and tumor cells plays a crucial role in tumorigenesis and progression. Tumor cells could promote glycolysis of CAFs, and alternatively, the CAFs could provide tumor cells with metabolites, such as pyruvate and lactate, through the OXPHOS and TCA cycle to fuel neighboring cancer cells. This phenomenon is known as the “Reverse Warburg Effect” [24, 44]. In fibroblasts activated by exosomes from human primary and metastatic colorectal cancer cells, caveolin-1 (Cav-1) expression was significantly down-regulated and GLUT1, the rate-limiting transporter of glucose uptake, was significantly up-regulated. Increased GLUT1 expression enhances glucose uptake and promotes glycolysis in activated fibroblasts [45]. Decreased Cav-1 expression significantly upregulates the expression of glycolytic enzymes such as lactate dehydrogenase and pyruvate kinase to promote glycolysis and promote cancer development [46]. Extracellular microRNAs (miRNAs) have recently been implicated in the intercellular crosstalk [47]. miRNA-105 in TDEs from breast cancer cells could enhance glutamine and glucose metabolism of CAFs to provide energy for neighboring tumor cells. When nutrient levels are low and metabolic by-products accumulate in the cells, CAFs are induced to overexpress the lactate transporter MCT4 (monocarboxylate transporter 4) to expel metabolic waste products lactate and β-HB from the cells. Lactate is utilized by MCT1-expressing cancer cells to increase OXPHOS levels [48, 49]. The metabolic symbiosis between CAFs and cancer cells requires that cells express the MCT1. Cancer cells induce HIF1a to create “pseudo-hypoxia” conditions for fibroblasts, and hypoxia induces calcium-dependent exosome release via MCT1 and CD147 to promote glycolysis [50, 51]. Exosomes miR-155 and miR-210 produced by melanoma are taken up by normal interstitial fibroblasts, resulting in decreased OXPHOS and increased aerobic glycolysis in fibroblasts, ultimately leading to extracellular acidification. At the same time, by inhibiting miRNA activity, exosome-mediated stromal cell metabolic reprogramming could be reversed. Matrix acidification promotes premetastatic niche formation through exosomes and promotes tumor migration and invasion [39]. Besides receiving exosomes from tumor cells, CAFs also secrete exosomes to act on cancer cells and regulate their metabolism. Uptake of CAFs derived exosomes in prostate and pancreatic cancer cells increases glutamine-dependent reductive carboxylation and glycolysis of cancer cells [52]. Through ^13^C metabolic flux analysis, cancer cells rapidly internalize CDEs, leading to depletion of extracellular exosomes within 24 h. Metabolites regulate glycolytic pathway flux through lactate supply and significantly alter intracellular metabolism within 24 h [53]. Thus, exosomes play an important role in the metabolic regulation interaction between tumors and CAFs.

TDE not only regulate the metabolism of CAFs, but also affect glucose metabolism of tumor-associated macrophages (TAM). Macrophages are one of the most abundant innate immune cells in TME and are associated with tumor growth, angiogenesis, metastasis, and poor prognosis. According to the surface markers of polarized macrophages and their functions, the polarized macrophages could be divided into two types: classically activated macrophages (M1) and alternatively activated macrophages (M2) [54]. The polarization of macrophage requires changes in intracellular metabolism. M1 requires less oxygen supplementation and has a high rate of glycolysis, which is conducive to survival in the hypoxic microenvironment of tumor and chronic inflammation sites. M2 is more likely to use OXPHOS and FAO (fatty acid oxidation) [55]. Jung et al*.* found that hypoxia-induced tumor cells could secrete more exosomes, affecting the recruitment of macrophages and promoting the polarization of M2 macrophages. This effect is achieved by TDEs that enhance oxidative phosphorylation of bone marrow-derived macrophages by transferring let-7a miRNA and inhibit the insulin-Akt-mTOR signaling pathway to affect macrophage metabolism [56]. In addition, TAMs show complex changes in metabolic patterns, including increased altered nitrogen cycling metabolism, glycolysis, and altered fatty acids synthesis, after being affected by different factors in TME [57]. These changes can induce host immunity evasion and promote tumor progression.

Tumor cells can systematically inhibit the use of nutrients by other cells to gain an advantage in growth and metastasis. For example, tumor cells secrete exosomes carrying high levels of miR-122 to inhibit glucose uptake by non-tumor cells in pre-metastatic sites [58]. Taken up by surrounding normal cells, miR-122 targets PKM2, a key mediator in the glycolytic pathway, and inhibits glycolytic metabolism, thereby reducing glucose utilization by niche non-tumor cells [58]. Metabolic remodeling of the premetastatic niche prior to the “arrival” of tumor cells has been recognized as an important means of promoting the sustained tumor progression and subsequent metastasis [59]. Fong et al*.* found that exosomal miR-122 secreted by breast cancer cells could inhibit glucose uptake by niche cells by down-regulating GLUT1 and PKM2. Inhibition of miR-122 in vivo can lower the glucose metabolism of distal organs such as brain and lung, and reduce the incidence of metastasis. This suggests that exosomal miR-122 can reshape whole-body energy metabolism and promote tumor progression [58].

Besides solid tumors, Wang et al*.* showed that exosomes secreted by acute myeloid leukemia (AML) cells, which contain VEGF/VEGFR, promote glycolysis in endothelial cells (ECs) [60]. Neovascularization provides oxygen and nutrients to tumor cells, supporting tumor development and providing a pathway for tumor metastasis. ECs in tumor vessels are more dependent on ATP produced by glycolysis than normal endothelial cells. Studies have shown that reducing high glycolysis in tumor ECs can induce therapeutic effects in preclinical tumor models [61].

Exosome mediated metabolic changes have been shown to affect the immune response to the tumor. For example, exosomes secreted by Pancreatic Ductal Adenocarcinoma (PDAC) cells which do not express SMAD can increase calcium flux and glycolysis by transferring differentially expressed proteins and miRNA associated with SMAD4. This transfer of exosomes has been shown to generate an immunosuppressive background [62]. It has been shown that overexpression of glycolytic molecules in tumor cells can impinge T-cell activation, while inhibition of glycolysis enhances T-cell-mediated antitumor immunity in vitro and in vivo [40]. Moreover, lactate, a glycolytic metabolite, has been shown to directly inhibit the cytolytic activity of NK cells and indirectly inhibit the function of NK cells by increasing the number of bone marrow derived suppressor cells (MDSCs) [63]. These findings suggest that immune suppression induced by metabolic recoding mediated by TDEs may be a potential trigger for tumor development.

### Lipid metabolism

Exosome-mediated disruption of lipid metabolism is increasingly recognized as a feature of tumor cells and may be a key factor in the progression and metastatic behavior of malignant tumors [64]. Studies have shown that lipid metabolism disorders can upregulate oncogenes such as Mtor, cyclin-E, c-Jun, Notch, c-Myb, and c-Myc to promote tumor invasion and metastasis [65]. Exosome membranes contain many molecules which may include phosphatidic acid (PA), phosphatidyl inositol (PI), phosphatidyl ethanolamine (PE), phosphatidyl choline (PC), phosphatidyl serine (PS), ceramide, cholesterol, sphingomyelin, glycosphingomyelin, and other lipids in low abundance. Some have suggested that PS and PE appear to be involved in the biogenesis of exosomes [66]. The enrichment of specific lipids has been shown to significantly increased exosome membrane hardness. Moreover, these lipids exist in the outer membrane of exosomes and play a crucial role in the recognition and internalization of exosomes, enabling them to deliver metabolites to recipient cell [67]. Depletion of a cholesterol lipid efflux pump ABCG1 (ATP-binding cassette transporter G1), leads to the accumulation of EVs and their derivatives, thereby triggering tumor regression [68]. Tafelmeier et al*.* have demonstrated that ABCG1-mediated cholesterol efflux promotes exosome release, while SRB1-mediated cholesterol efflux inhibits exosome uptake by recipient cells [69].

The role of lipids in cell communication is an interesting emerging topic of research and is worth further investigation. Exosomes are known to carry bioactive lipids, such as prostaglandins and leukotrienes, which have been shown to promote the development of tumors [70]. Furthermore, Lydic et al. found that TDEs from colorectal cancer cell line LIM1215 have higher levels of glycerolipids, cholesterol, glycerol, and sphingolipids [27]. Others have shown that TDEs from prostate cancer cells are rich in phosphatidylserine, glycosphingolipids, sphingomyelin, and cholesterol [28]. High fat content appears to be more conducive to the uptake of TDEs by normal cells, inducing the transformation of normal cells into tumor cells [65, 71, 72].

It has been shown that tumor derived signaling molecules trigger lipolysis in cancer-associated adipocytes (CAAs), which results in lipoatrophy in humans [73], a form of cancer cachexia [74]. Phospho-hormone-sensitive lipase (P-HSL), a marker that activates lipolysis, was found at higher levels in TDEs from Lewis lung Cancer (LLC). Adipocytes exposed to TDEs from LLC showed lower levels of lipid droplets and higher levels of glycerol release [75]. Another study showed that TDEs from pancreatic cancer cells containing adrenomedullin (AM) interact with adrenomedullin receptors (ADMRs) in adipocytes and activate ERK 1/2 and MAPKs p38 signaling pathways to induce lipolysis via HSL phosphorylation [76]. Wang et al. found that TDEs from lung cancer could be internalized by human adipose tissue-derived MSCs and participate in the inhibition of adipogenesis of MSCs through TGFβ signaling pathway [77]. The effect between the two is reciprocal, TDEs regulate the metabolism of MSCs, and MSCs affected by TDEs secrete more exosomes as a kind of feedback to promote tumor angiogenesis [78]. Whether MSC-derived EVs promote or inhibit cancer seems to depend on the contents of cytokines and miRNA in exosomes [79–81]. Interestingly, lipids carried in exosomes have also been found to be important for inducing tumor drug resistance. For example, through regulating lipid metabolism, studies have shown that high expression of acid sphingomyelinase (ASM) by multiple myeloma (MM) derived exosomes can transfer drug-resistant phenotypes to drug-sensitive MM cells. The expression and protein level of ASM in MM cells and exosomes increased after antitumor drug stimulation, reflecting the tumor protective effect of ASM and promoting the occurrence of drug resistance [82].

Lipids in TDEs have also been shown to alter immune responses. For example, Jiang et al*.* found that overexpression of FASN (Fatty Acid Synthase) in ovarian cancer led to lipid accumulation in TME, resulting in T cell dysfunction, and then impaired anti-tumor immune responses [83]. It has also been shown that high cholesterol is more conducive for exosomes to bind to CD8 ( +) T cells, as the enrichment of cholesterol in cell membranes can improve the fluidity of cell membranes [84].

### Amino acid metabolism and nucleotide metabolism

Studies on exosomes and amino acid metabolism mainly focus on tumor cells and CAFs and provides evidence that TDEs could induce CAFs production [85]. Liu et al*.* found that CAFs mainly regulated amino acid metabolism in an exosome-dependent manner in lung adenocarcinoma (LUAD) cells. Stimulated by tumor-derived proinflammatory cytokines, the specific long noncoding RNA LINC01614 secreted from CAFs, was up-regulated. CAF-derived exosomes could promote NF-κB activation through transport of LINC01614 to LUAD cells, which then interacted with ANXA2 and p65, leading to upregulation of glutamine transporters SLC7A5 and SLC38A2. Ultimately, LINC01614 enhanced glutamine uptake in LUAD cells [86]. Moreover, Zhao et al. found that exosomes secreted by CAFs could significantly inhibit electron transport chains after being absorbed by prostate and pancreatic cancer cells, thus increasing glutamine dependent reduction carboxylation [52]. The above studies suggest that exosomes play a key role in regulating the metabolism of glutamine. Furthermore, because glutamine is a nitrogen donor for nucleotide synthesis [87] TDEs effects on glutamine is likely to also affect nucleotide synthesis. Therefore, it could be speculated that TDEs may regulate the generation and degradation of nucleic acids by reacting to nucleotide metabolites and metabolic wastes. While the effects of TDEs on nucleotide metabolism is interesting, the current understanding about this biology is limited and further studies are warranted.

## Effects on systemic metabolism

The normal state of the body is one of equilibrium and homeostasis. The development of neoplasms can upset this balance, for example emaciation is considered to be a manifestation of tumor cachexia. It has been shown that TDEs from pancreatic cancer could induce subcutaneous adipose tissue lipolysis through AM as a mediator, suggesting that TDEs induced lipolysis may be associated with weight loss in patients [76]. In addition, Fong et al*.* found that inhibition of exosomal miR-122 could normalize glucose metabolism of the brain, lung and other distal organs and reduce the incidence of tumor metastasis, indicating that exosomal miR-122 could reshape the whole body energy metabolism and promote tumor progression [58]. It has been found that TDEs from prostate cancer (PCa) containing PKM2 could be transferred to bone marrow stromal cells (BMSCs) and promote tumor metastasis by altering bone metabolism [88]. And It has been found that TDEs from PCa transfer LncRNA nuclear-enriched abundant transcript 1 (NEAT1) to human bone marrow-derived MSCs promote osteogenic differentiation, suggesting that this might be one of the reasons why patients with PCa often present with osteoblast bone metastases [89]. In summary, TDEs have been shown to have significant effects on the whole-body metabolism, although the whole-body metabolism is complex, and requires further exploration.

Recent studies have found that metabolism alterations caused by TDEs can affect the immune system which can aid in the ability of the tumor to escape the immune system. Studies have shown that TDEs can lead to increased glucose uptake by macrophages in the pre-metastasis niche through TLR2 and NF-κB signaling pathways, thus increasing the expression of PD-L1 and promoting the polarization of macrophages towards immunosuppressive phenotypes [90]. In addition, TDEs activate Tregs cells to form immunotolerant premetastatic niches by regulating the interaction of CCL1 + fibroblasts and CCR8 + Treg cells [91]. This provides immune conditions for tumor metastasis. Peroxisome proliferator activated receptor (PPAR) α response to fatty acids delivered by TDEs and leads to excess lipid droplet formation and enhanced fatty acid oxidation, ultimately leading to metabolic shift to mitochondrial oxidative phosphorylation, which drives dendritic cell immune dysfunction [92]. In summary, multiple studies have shown that TDEs mediated metabolism alterations can cause deleterious effects for the ability of the immune system to target tumor cells.

## Application

### Diagnostic biomarkers

**Fig. 2:**
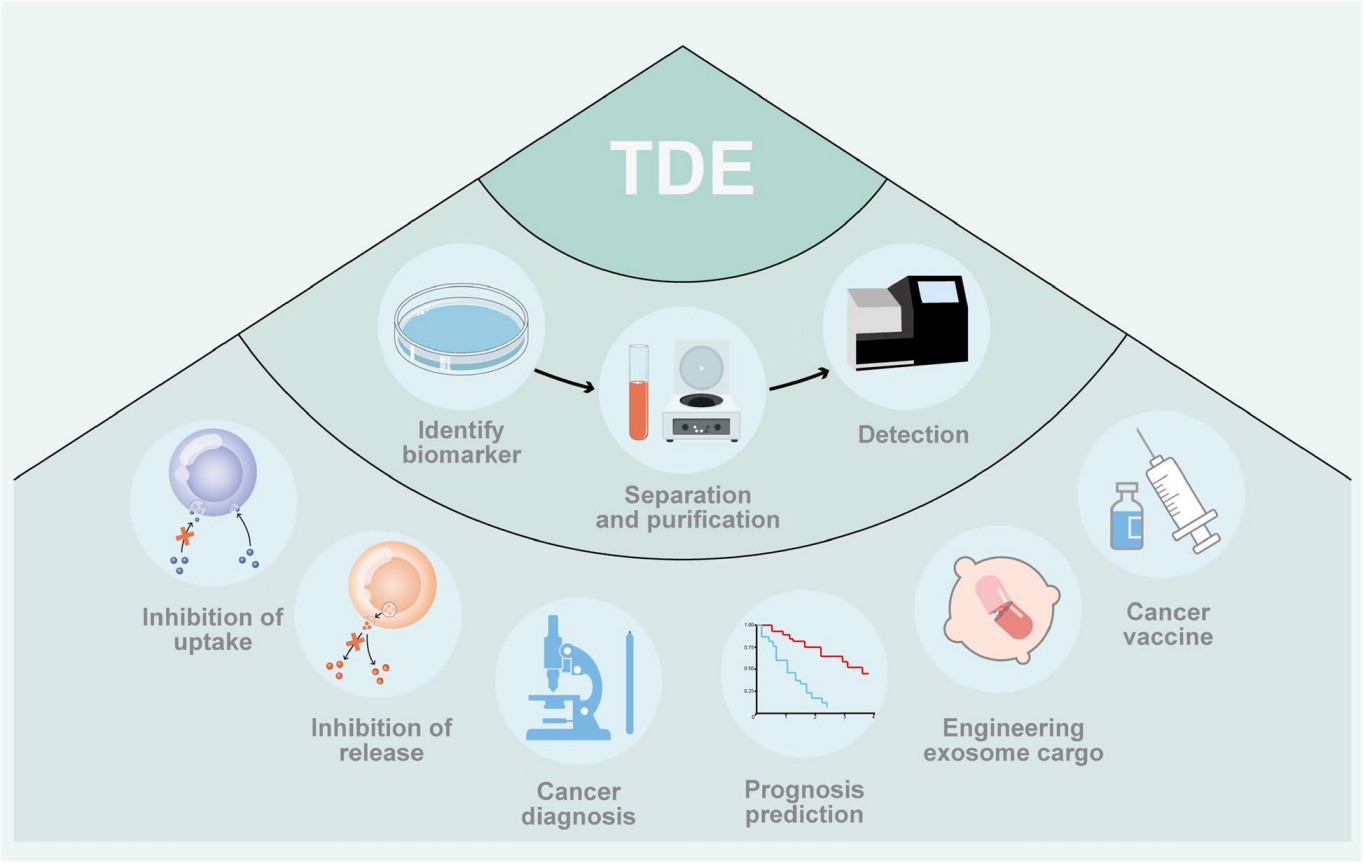
The application of tumor-derived exosomes (TDEs). The application of tumor-derived exosomes (TDEs) is mainly divided into two kinds: as a biomarker for diagnosis and participating in treatment. Starting from the diagnostic biomarkers, the biomarkers related to diagnosis or prognosis should be determined first, and then the isolated and purified exosomes can be obtained by centrifugation, filtration and other methods, and substances required for detection in the exosomes can be detected to help diagnosis and treatment. From the perspective of treatment, the tumor-promoting function can be blocked by inhibiting the release of TDEs or inhibiting the absorption of TDEs by other cells. TDEs could also be used to load drugs or prepare tumor vaccines

A large number of tumor markers carried by cancer cell exosomes have set off a boom in liquid biopsy. The application of TDEs was shown in Fig. 2. Depending on the chemical, physical, and biological properties of exosomes, different methods have been used to isolate and purify exosomes, including ultra-centrifugation, size exclusion chromatography, ultrafiltration, and microplate-based magnetic immunocapture. Using multiple techniques based on membrane protein composition, size and density, rich exosome populations could be isolated from the various biological fluids and cell media [93]. The liquid biopsy strategy assesses factors including circulating tumor cells, circulating tumor DNA (ctDNA), EVs or exosomes, and other biochemical substances [94–96]. Since exosomes are detectable in almost all body fluids, they are more readily available, making them ideal biomarkers for monitoring dynamic intratumoral heterogeneity (ITH), enabling early detection and minimizing treatment side effects and toxicity [22, 97]. There has been a growing number of clinical trials related to exosomes in multiple cancer types, a summary of these trials are shown in Table 1.

**Table 1:** Ongoing clinical trials of exosomes in cancer diseases

Type of tumour	Purpose	Details*			
		**NCT number**	**Start date**	**Primary objective of exosome analysis**	**Phase**
**Breast Cancer**	Biomarker	NCT04781062	January 19, 2021	Early detection of breast cancer	-
		NCT05286684	January 4, 2023	Diagnosis of metastatic meningitis	-
		NCT04288141	December 20, 2019	Measure the Expression of the HER2-HER3 Dimer	-
		NCT04258735	July 17, 2019	Genomic profiling	-
**Digestive and gynecological / breast cancers**	Biomarker	NCT04530890	January 20, 2021	Diagnostic and prognostic biomarkers	-
**Cholangiocarcinoma**	Biomarker	NCT03102268	May 1, 2017	Exosomes profiling	-
**Hepatocellular Carcinoma**	Therapy	NCT05375604	June 28, 2022	Macrophage Reprogramming Agent, exoASO-STAT6 (CDK-004)	Phase 1
**Gallbladder Carcinoma**	Biomarker	NCT03581435	January 1, 2018	Exosomes profiling	-
**Clear Cell Renal Cell Carcinoma**	Biomarker	NCT04053855	January 29, 2020	CA9 + and CD9 + exosome analysis	-
**Bladder Cancer**	Biomarker	NCT05270174	June 1, 2023	Preoperative diagnosis of lymphatic metastasis using urinary exosome lncRNAs	-
	Therapy	NCT05559177	September 1, 2022	Chimeric Exosomal Tumor Vaccines	Phase 1
**Colorectal Cancer**	Biomarker	NCT04394572	January 7, 2021	Diagnostic biomarkers	-
		NCT03874559	February 13, 2018	Exosomes profiling	-
		NCT04523389	July 1, 2020	Prognostic biomarkers	-
		NCT04227886	December 1, 2019	Predictive biomarkers	-
		NCT04852653	September 21, 2021	Predictive biomarkers	-
	Therapy	NCT01294072	January 2011	Plant Exosomes combined with curcumin for colon cancer	Phase 1
**Gastric Cancer**	Biomarker	NCT05334849	November 1, 2018	Exosomal lncRNA-GC1 detection	-
		NCT05397548	May 1, 2022	Exosomal lncRNA-GC1 detection	-
		NCT05427227	July 1, 2022	Predictive biomarkers	-
**Head and Neck Cancer**	Therapy	NCT01668849	August 2, 2012	Plant Exosomes to treat oral mucositis	Phase 1
**Lung Cancer**	Biomarker	NCT03542253	May 20, 2018	Diagnostic biomarkers	-
		NCT04529915	April 9, 2020	Diagnostic biomarkers	-
		NCT04939324	June 21, 2021	Exosomes molecular Profiling	-
		NCT04629079	October 23, 2020	Diagnostic biomarkers	-
		NCT04323579	July 1, 2018	Diagnostic biomarkers	-
		NCT03830619	January 1, 2017	Diagnostic biomarkers	-
		NCT04315753	January 1, 2018	Diagnostic biomarkers	-
		NCT05218759	February 21, 2022	Predictive biomarkers	-
		NCT05424029	June 14, 2022	Prognostic biomarkers	-
		NCT04427475	June 8, 2020	Predictive biomarkers	-
		NCT03317080	September 27, 2017	Prognostic and predictive biomarkers	-
		NCT03108677	May 1, 2017	Diagnostic and prognostic biomarkers	-
		NCT04182893	October 8, 2019	Diagnostic biomarkers	-
		NCT04499794	August 1, 2020	Diagnostic and predictive biomarkers	-
		NCT03228277	July 17, 2017	Predictive biomarkers	Phase 2
	Therapy	NCT01159288	May 19, 2010	Exosomal Tumor Vaccines	Phase 2
**Melanoma**	Biomarker	NCT02310451	December 2014	Exosomes profiling	-
**Non-Hodgkin B-cell Lymphomas**	Biomarker	NCT03985696	July 2, 2019	Exosomes profiling and diagnostic function	-
**Osteosarcoma**	Biomarker	NCT05101655	October 1, 2020	Diagnostic and predictive biomarkers	-
**Ovarian Cancer**	Biomarker	NCT03738319	November 10, 2018	Exosomes profiling	-
**Pancreatic Cancer**	Biomarker	NCT03821909	August 1, 2018	Exosomes profiling and diagnostic function	-
		NCT02393703	March 2015	Exosomes profiling	-
		NCT03791073	January 1, 2018	Exosomes profiling	-
		NCT03032913	February 15, 2017	Diagnostic biomarkers	-
		NCT04636788	November 1, 2020	Diagnostic and prognostic biomarkers	-
		NCT05441189	April 19, 2021	Prognostic and predictive biomarkers	-
	Therapy	NCT03608631	January 27, 2021	Mesenchymal Stromal Cells-Derived Exosomes with KrasG12D siRNA	Phase 1
**Prostate Cancer**	Biomarker	NCT02702856	May 2014	Diagnostic biomarkers	-
		NCT04556916	February 19, 2021	Diagnostic biomarkers	-
		NCT03694483	October 3, 2018	Diagnostic biomarkers	-
		NCT03236688	February 2016	Exosomes profiling	-
		NCT04720599	June 1, 2020	Diagnostic biomarkers	-
		NCT03911999	May 3, 2018	Prognostic exosomes	-
**Thyroid Cancer**	Biomarker	NCT03488134	August 3, 2018	Prognostic biomarkers	-
		NCT02862470	August 5, 2016	Prognostic biomarkers	-
		NCT03109847	January 5, 2017	Exosomes profiling	-
		NCT05463107	August 1, 2022	Diagnostic biomarkers	-
		NCT04948437	August 19, 2021	Prognostic biomarkers	-
**Bone Metastases**	Biomarker	NCT03895216	December 3, 2018	Prediction of bone metastasis	-

^*^These clinical trials are from ClinicalTrials.gov. (https://clinicaltrials.gov/)

Although the development of science and technology for exosome detection is growing, there are still many problems to be solved. First, each exosome assay technique is known to have its own bias in estimating exosome size. For example, Nanoparticle Tracking Analysis (NTA) is widely used for exosome size detection in bioparticle applications, and its detection limit for bioparticles is about 70 nm. NTA and Transmission Electron Microscopy are sensitive to different sizes [98]. Moreover, the process of exosome preparation may result in swelling, shrinkage, or obesity of the exosome, and these changes have a significant impact on true size analysis [66]. In addition, due to technical limitations, it is not possible to obtain fully purified exosomes, which also affects the accuracy of EV analysis. For example, EVs in the typical exosome size range are known to include exosomes (20—100 nm), microvesicles (100–1000 nm) and apoptotic bodies (1–5 μm), VLDL, chylomicrons, and retroviruses cannot be effectively separated from exosomes completely by centrifugation because of their similar membrane orientation and density [99]. Furthermore, Exosome size may vary considerably even within the same single cell line, perhaps due to the inhomogeneous invagination inherent in the restrictive membrane during exosome biogenesis [66]. Interestingly, exosome size has been reported to be related with certain diseases. For example, in patients with NSCLC, tumor-draining pulmonary vein blood secrete body size (< 112 nm) is associated with a shorter time to recurrence and shorter overall survival [100].

The exosome population is made up of exosomes of different internal and external carriers, different sizes, different cellular origins, different functional effects on the recipient cell, and resulting in uneven application functions [66]. The content of exosomes reflects the state of secretory cells to some extent, making them potentially useful for assessing normal and pathophysiological status [3, 101].

Recent studies have shown that exosomes can be used as biomarkers to diagnose, identify the stage and the subtype of tumor cells. In 2015, Dr. Raghu Kallur’s team found that GPC1 protein contained in exosomes of pancreatic cancer cells could be used as a non-invasive method to diagnose and screen early pancreatic cancer at a stage suitable for surgical treatment. Most importantly, it can distinguish chronic pancreatitis from early or advanced pancreatic cancer, providing a new approach to the diagnosis of early pancreatic cancer, which is difficult to detect clinically [102]. Moreover, through the bone marrow, PCa cells create a pre-metastatic niche through primary PCa TDEs mediated PKM2 transfer to BMSCs and subsequent CXCL12 upregulation. This novel mechanism suggests that exosomal PKM2 may serve as a therapeutic biomarker target for PCa bone metastases [88]. Furthermore, Roberg-Larsen et al*.* showed the TDEs from MCF-7 cell line (estrogen receptor (ER +) breast cancer cell line) had increased levels of 27-OHC in compared with TDEs from the ER- breast cancer cell line (MDA-MB-231), providing evidence that TDEs may contain additional information of diagnostic value [103].

Exosomes are stable sources of miRNA in body fluids, which prevent the degradation of biomacromolecules under fluctuating body conditions [104]. These highly stabile miRNAs in exosomes are attractive non-invasive biomarker targets for cancer screening and disease surveillance. Analysis of exosome miRNAs in the sera of healthy individuals and cancer patients revealed important differences related to tumor progression, while highlighting the potential value of these miRNAs as biomarkers of disease prognosis [105]. miRNA detected in serum TDEs from breast cancer patients can be used to discriminate between specific molecular subtypes. Furthermore, it has been found that high level of miR-373 in TDEs from breast cancer correlates with triple-negative or other highly aggressive breast cancer types, highlighting the potential role of serum-specific exosomal miR-373 as an aggressive tumor biomarker [106]. The identification of in vitro miRNAs that associate with tumor metastases could provide an additional diagnostic tool to assess disease stage and monitor its progression. High levels of miR-105 have been found in serum-derived exosomes from breast cancer patients who later develop metastatic disease [107]. Similarly, down-regulation of miR-19a and/or miR-29c and up-regulation of miR-210 have been detected in TDEs from brain metastatic breast cancer cells [108]. The overexpression of miR-483-3p occurs in the early development of PDAC and exists in precancerous PanIN lesions, providing evidence that miR-483-3p might be a biomarker for early diagnosis and prognosis of PDAC [109].

### Treatment

One interesting therapeutic idea involves harnessing the functionality of exosomes, which transport proteins, lipids, and nucleic acids to mediate cell-to-cell communication between TMEs components, for anticancer therapy. Therefore, research focused on targeting exosome biogenesis and loading is required before the exosome could be used as a viable strategy to treat cancers.

Recent studies have investigated if manipulating the release of TDEs could be used for therapeutics. Inhibition of Rab27 with targeted shRNAs has been shown to reduce exosome release, but this manipulation also leads to a significant increase in smaller endosome-sized vesicles (50 nm), implying that Rab proteins have the ability to alter the size distribution of exosomes [110]. Others have shown that GW4869 (exosome-release inhibitor) can block the secretion of exosomes and reverse metabolic changes in breast cancer cells. GW4869 inhibits glycolysis and receptor cell activation in tumor cells, thereby inhibiting cancer progression [111, 112]. Fanny et al*.* found that reducing exosomes production with dimethyl amiloride enhanced the antitumor efficacy of the chemotherapy drug cyclophosphamide in vivo in three different mouse tumor models [113]. Another possible mechanism to inhibit the tumor-promoting function of TDEs is to prevent exosome fusion or uptake by target cells. One study suggested that TDEs uptake by cells could be prevented by targeting specific exosome biomarkers [114]. For example, a recent study showed that positively charged mesoporous silica nanoparticles (MSNs) with EGFR-targeting aptamers (MSN-AP) could interact and eliminate circulating cancer-derived negatively charged exosomes by allowing them to enter the small intestine, thereby reducing metastasis formation [115].

Exosomes have also been used to build cancer vaccines. For example, Huang et al*.* loaded Hiltonol (TLR3 agonist) and the immunogenic cell death inducers human neutrophil elastase into α-LA (α-lactalbumin)-engineered breast cancer-derived exosomes to form an in situ DC vaccine (HELA-Exos). HELA-Exos has been shown to exhibit potent antitumor activity in both mouse models and human breast cancer organoids, which improves subsequent tumor-reactive CD8 + T-cell response by promoting the activation of type one conventional dendritic cells in situ [116]. Taking advantage of the property that TDEs can specifically deliver drugs to the tumor site, Zhu et al*.* found that TDEs could be developed for combined delivery of AIEgen and proton pump inhibitors (PPI) for the combined treatment of gastric cancer [117]. In addition, a recent study found successful evaluation of drug efficacy by using exosome-synthesized probes to detect drug occupancy [118]. While the early studies of exosome cancer vaccines are promising, more researches are needed to determine the feasibility of antitumor exosome for use in human and mass production. In addition to using cell exosomes, artificial engineering such as freeze–thaw cycles, electroporation, ultrasound, reagent transfection, or saponin methods, are also methods for loading drugs or functional cargo into exosomes [119]. Hypoxia might influence the suitability of exosome cargo as a scaffold for fusion of functional molecules and other drugs, thus affecting the efficiency of treatment. In addition, exosomes in TME exhibit specific uptake under hypoxic conditions, which might provide a pathway for specific targeting of malignant tumors [22].

## Conclusion and prospects

Tumor-derived exosomes play a key role in the development of tumors. By regulating the glucose and lipid metabolism of tumor cells and other cells in TME, TDEs promotes more suitable soil and materials for tumor growth. From this point of view, many researchers hope to provide new strategies for the diagnosis and treatment of cancer patients by improving the technology of exosomes detection and isolation, inhibiting the secretion of TDEs, or blocking the binding of TDEs with targeted cells. However, there are also problems that need attention, including how to quickly and effectively load drugs into exosomes, how to enhance the stability, and how to improve the specificity and targeting. It is believed that in the near future, with the progress of science and technology and the continuous efforts of researchers, these questions will get addressed, providing new benefits to patients.

## Data Availability

Not applicable.

## Abbreviations

EVs: Extracellular vesicles
TDE: Tumor-derived EVs
ILVs: Intraluminal vesicles
MVBs: Multivesicular bodies
ESCRT: Endosomal sorting complex required for transport
ARF6: ADP ribosylation factor 6
PLD2: Phospholipase D2
TCA: Tricarboxylic acid cycle
OXPHOS: Oxidative phosphorylation
TME: Tumor microenvironment
NSCLC: Non-small cell lung cancer
MSCs: Mesenchymal stem cells
PKM2: Pyruvate kinase M2
SNAP-23: Synaptosome associated protein 23
PtNPs: Platinum nanoparticles
CAFs: Cancer associated fibroblasts
ECM: Extracellular matrix
Cav-1: Caveolin-1
MCT4: Monocarboxylate transporter 4
TAM: Tumor-associated macrophages
FAO: Fatty acid oxidation
ECs: Endothelial cells
PDAC: Pancreatic Ductal Adenocarcinoma
MDSCs: Marrow derived suppressor cells
PA: Phosphatidic acid
PI: Phosphatidyl inositol
PE: Phosphatidyl ethanolamine
PC: Phosphatidyl choline
PS: Phosphatidyl serine
ABCG1: ATP-binding cassette transporter G1
CAAs: Cancer-associated adipocytes
P-HSL: Phospho-hormone-sensitive lipase
ADMRs: Adrenomedullin receptors
ASM: Acid sphingomyelinase
FASN: Fatty Acid Synthase
LUAD: Lung adenocarcinoma
PCa: Prostate cancer
BMSCs: Bone marrow stromal cells
NEAT1: Nuclear-enriched abundant transcript 1
ctDNA: Circulating tumor DNA
ITH: Intratumoral heterogeneity
NTA: Nanoparticle Tracking Analysis
MSN-AP: Mesoporous silica nanoparticles with EGFR-targeting aptamers
